# Inhibition of Aurora B kinase (AURKB) enhances the effectiveness of 5-fluorouracil chemotherapy against colorectal cancer cells

**DOI:** 10.1038/s41416-024-02584-z

**Published:** 2024-01-29

**Authors:** Esha T. Shah, Christopher Molloy, Madeline Gough, Thomas Kryza, Selwin G. Samuel, Amos Tucker, Maneet Bhatia, Genevieve Ferguson, Rebecca Heyman, Shivam Vora, James Monkman, Emma Bolderson, Arutha Kulasinghe, Yaowu He, Brian Gabrielli, John D. Hooper, Derek J. Richard, Kenneth J. O’Byrne, Mark N. Adams

**Affiliations:** 1 Centre for Genomics and Personalised Health, School of Biomedical Sciences, Faculty of Health, Queensland University of Technology, Translational Research Institute, 37 Kent Street, Woolloongabba, QLD 4102 Australia; 2 Mater Research Institute – The University of Queensland, Translational Research Institute, 37 Kent Street, Woolloongabba, QLD 4102 Australia; 3 Frazer Institute, Faculty of Medicine, The University of Queensland, Translational Research Institute, 37 Kent Street, Woolloongabba, QLD 4102 Australia; 4 Cancer Services, Princess Alexandra Hospital, Ipswich Road, Woolloongabba, QLD 4102 Australia

**Keywords:** Colorectal cancer, Cancer therapeutic resistance

## Abstract

**Background:**

5-Fluorouracil (5-FU) remains a core component of systemic therapy for colorectal cancer (CRC). However, response rates remain low, and development of therapy resistance is a primary issue. Combinatorial strategies employing a second agent to augment the therapeutic effect of chemotherapy is predicted to reduce the incidence of treatment resistance and increase the durability of response to therapy.

**Methods:**

Here, we employed quantitative proteomics approaches to identify novel druggable proteins and molecular pathways that are deregulated in response to 5-FU, which might serve as targets to improve sensitivity to chemotherapy. Drug combinations were evaluated using 2D and 3D CRC cell line models and an ex vivo culture model of a patient-derived tumour.

**Results:**

Quantitative proteomics identified upregulation of the mitosis-associated protein Aurora B (AURKB), within a network of upregulated proteins, in response to a 24 h 5-FU treatment. In CRC cell lines, AURKB inhibition with the dihydrogen phosphate prodrug AZD1152, markedly improved the potency of 5-FU in 2D and 3D in vitro CRC models. Sequential treatment with 5-FU then AZD1152 also enhanced the response of a patient-derived CRC cells to 5-FU in ex vivo cultures.

**Conclusions:**

AURKB inhibition may be a rational approach to augment the effectiveness of 5-FU chemotherapy in CRC.

## Introduction

Colorectal cancer (CRC) is the second leading cause of cancer-related mortality and the third most diagnosed cancer worldwide [1]. More than 900,000 deaths were recorded and approximately 1.9 million cases were diagnosed in 2020 [2]. CRC is typically diagnosed within the older population and has a 5-year survival rate of 65% [3]. Current treatment options for CRC include surgery, chemotherapy, radiotherapy, and targeted therapy [4].

Chemotherapy remains a mainstay therapy for CRC. Standard-of-care front-line chemotherapy includes 5-fluorouracil (5-FU), or capecitabine the oral prodrug-form of 5-FU, in combination with oxaliplatin, or irinotecan. 5-FU is a uracil analogue that is converted into three primary active metabolites: fluorodeoxyuridine monophosphate (FdUMP), fluorodeoxyuridine triphosphate (FdUTP) and fluorouridine triphosphate (FUTP) [5]. The primary mechanism of each metabolite is the inhibition of the nucleotide synthesis enzyme, thymidylate synthase eventually resulting in DNA damage and cell death [6]. Additional mechanisms of cytotoxicity comprise the incorporation of 5-FU derivatives into RNA and DNA. Misincorporation of FdUTP into cellular DNA results in significant impairment of DNA repair mechanisms. The combined effects exerted by 5-FU metabolites contribute to the promising responses observed during initial stages of 5-FU treatment [6]. However, despite its multiple mechanisms of action, tumour cells gradually develop resistance to 5-FU rendering the therapy ineffective [7].

Several mechanisms mediating resistance to 5-FU have been postulated. For example, most notably the ability of thymidine kinase to salvage thymidylate from thymidine, enabling DNA replication and repair of damaged cells and thus, cancer progression. Another issue encountered is the extensive metabolism of 5-FU by dihydropyridine dehydrogenase (DPD), before it enters the cell [6]. The high abundance of DPD expressed in the liver means that only 3% of the administered dose of 5-FU remains active and therefore, capable of mediating cytotoxic, anti-tumour effects [6]. Further research is needed to develop combinatorial strategies to augment the therapeutic effectiveness of chemotherapy and prevent or delay the emergence of resistance to 5-FU.

In the present study, we have sought to identify unique strategies to improve sensitivity to 5-FU. Augmenting the therapeutic effect of chemotherapy, might be a strategy to improve response to therapy and enhance health outcomes for people living with CRC. As such, we employed quantitative proteomics to identify proteins and molecular pathways that are differentially regulated in response to 5-FU. With this approach, we sought to determine whether the identified deregulated proteins and pathways were druggable to improve sensitivity to 5-FU. Of the proteins identified within our study, we demonstrate that Aurora kinase B (AURKB) is upregulated following a 24 h treatment with 5-FU and at the core of a network of upregulated proteins. As a druggable kinase, we further demonstrate using in vitro and ex vivo models of CRC that inhibition of AURKB with the small molecular weight prodrug AZD1152 enhanced sensitivity to 5-FU. The results suggest that inhibition of AURKB may be a rational strategy to improve the effectiveness of 5-FU that warrants further evaluation.

## Materials and methods

### Antibodies and reagents

The pH3Ser10 antibody (#53348) and p53 (DO-7) antibody (#48818) were purchased from Cell Signalling Technology (Genesearch, Gold Coast, Australia). The anti-Aurora B antibody (611082) was from BD Transduction Laboratories while the anti-γH2AX antibody (ab26350) was from Abcam. Donkey anti-rabbit and anti-mouse Alexa Fluor 488 antibodies were purchased from Thermo Fisher Scientific (Scoresby, Australia). AZD1152 was purchased from Selleck Chemicals Llc (Sapphire Bioscience, Redfern, Australia). All other reagents were purchased from Sigma-Aldrich (Castle Hill, Australia) except where noted.

### Cell culture and drug treatment

All cell lines were purchased from ATCC (Manassas, VA, U.S.A.), screened for mycoplasma contamination and maintained at 37 °C in a humidified atmosphere containing 5% CO_2_ in DMEM medium supplemented with 10% (v/v) heat-inactivated fetal bovine serum (FBS).

Prior to differential mass spectrometry-based quantitative proteomics, HCT116 cells were treated with 50 µM 5-FU for 24 h. For drug potency experiments (see cell viability assays section), cells were treated with increasing concentrations of 5-FU (5 µM to 1 mM) for 24 h, followed by treatment with AZD1152 (50 nM or 200 nM) for 24 h in fresh culture media. For all other in vitro 2D and ex vivo drug treatments, cells were treated with 50 µM 5-FU for 24 h followed by treatment with the absence or presence of AZD1152 (50 nM or 200 nM) in fresh culture media. For 3D and ex vivo experiments, drug treatment concentrations are as indicated.

### Nano liquid chromatography mass spectrometry analysis

Mass spectrometry (MS) analysis was performed as previously described [8], using an AB Sciex 5600+ TripleTOF mass spectrometer interfaced to an Ekspert^TM^ NanoLC system.

For analysis and statistical testing, log2 transformation was first performed and differentially regulated proteins identified by applying empirical Bayes moderated t-statistics tests [9, 10] using the R statistical environment (version 3.5.2) and application of Benjamini-Hochberg correction [11] to control the false discovery rate (FDR (or *q*-value)). The list of all quantified proteins (including differentially regulated proteins) by LC-MS/MS are listed in Supplemental Table 1.

### Immunofluorescence, high content imaging, live cell imaging and analysis

High content immunofluorescence and imaging for mitotic index and DNA damage response was performed as previously described [12]. Briefly, cells seeded in glass bottom 96-well plates were fixed with 4% paraformaldehyde for 20 min at ambient temperature then permeabilized with 0.1% Trion X-100 in PBS for 5 min. Cells were blocked with 2% donkey serum in PBS before incubation with an anti-pH3Ser10 antibody overnight at 4 °C, used at a dilution of 1:1000. Alexa Fluor® secondary antibodies were incubated for 1 h at ambient temperature in 0.5% donkey serum in PBS followed by staining with DAPI. Images were collected using an InCell Analyser 6500 high content microscopy system (GE Healthcare Life Sciences, Paramatta, Australia). Mitotic index and γH2AX foci per nuclei were calculated from images using the CellProfiler software v3.1.9 and reported as either percentage of cells positive for pH3Ser10 staining per field of view, or number of γH2AX foci/nuclei per field of view from a minimum of 1100 nuclei over three independent experiments.

Flow cytometry to examine cell cycle stage by DNA content was performed as previously described [13]. Briefly, lifted cells were fixed with 70% ethanol and stained in PBS containing propidum iodide and RNaseA for 1 h. Cells were analysed using a Beckman Coulter CytoFLEX-S flow cytometer and the FlowJo X software.

For live cell imaging, cells seeded in glass bottom 24-well plates were drug treated and imaging was immediately commenced over the course of 72 h on a Zeiss AxioObserver 7 microscope. Cells were incubated within a humidification chamber on the microscope. Analysis was performed using Zen 3.2 acquisition and analysis software.

### Cell viability assays

Cells were seeded into a white-walled, glass-bottom 384-well plate (Nunc) at a density of 1 × 10^3^ cells per well. 24 h following seeding, the cells were treated with escalating concentrations of 5-FU alone or for the sequential combination approach, a low and high concentration of AZD1152 was added 24 h following treatment with 5-FU. Cell viability was determined using CellTitre-Glo 2.0 (Promega Corporation), according to the manufacturer’s instructions, 72 h following commencement of drug treatments. Luminescence was measured and analysed on the FLUOstar Omega Microplate Reader (BMG Labtech). Data was normalised to untreated controls, and dose response curves and drug potency values generated using GraphPad Prism V9 software.

### 3D models

HCT116 and HT29 cells were seeded (1 × 10^4^ per well) in an ultra-low attachment 96-well plate (Corning) with 1× DMEM Happy Cell® ASM (Vale Life Sciences), following the manufacturer’s instructions. Cultures were maintained for 72 h following which, the spheroids were treated with 5-FU or AZD1152 alone, or the sequential combination of both drugs over a total period of 96 h. Multiple *z*-stack images were taken using an InCell Analyser 6500 high content microscopy system and equatorial plane spheroid area calculated using the open-source ImageJ analysis as previously described [14–16].

### Patient derived xenografts

Fresh colorectal cancer specimens were used to generate patient derived xenografts (PDXs) in NOD.Cg-*Prkdc*^*scid*^ *Il2rg*^*tm1Wjl*^/SzJ (NSG) mice as previously described [17], with written informed consent from the patients, under the approval of the Wesley Hospital Human Research Ethics Committee. PDXs were maintained following the established protocol and used for experiments within 5 generations of passaging in mice which were ensured with consistent histology with the original patient tumours [17]. Experiments involving mice were approved by the University of Queensland Animal Ethics Committee.

### Ex vivo culture model

Fresh PDXs were harvested from mice and immediately sectioned for explant experiments. A Vibratome VT1200 (Leica Microsystems) was used to cut thin (300 µm) slices from the colorectal PDX tumour sample. Tumour samples were orientated, embedded in 5% low melt agarose, placed on ice to set, and immobilised on a specimen disk using cyanoacrylate glue. Sectioning was performed with ice-cold sterile balanced salt solution in the buffer tray to assist in tissue sectioning and collection. Slicing speed was set at 0.6 mm/s and vibration amplitude at 3.0 mm [18]. Three tumours grown from individual mice were employed for this experiment.

Tissue slices were cultured in sextuplicate in wells of a 12-well plate, subjected to 60 rpm using the Stuart SSM1 mini orbital shaker placed in an incubator. Tissue culture was performed at 37 °C in a 5% CO2 using 3 ml/well of Phenol-Free Red RPMI (Gibco) supplemented with 10% heat-inactivated FCS, 200 mM Glutamine, 1X penicillin streptomycin, insulin (10 μg/ml) and hydrocortisone (10 μg/ml). Explant slices were cultured in media overnight, followed by respective treatments for 72 h. Treatments included were vehicle (DMSO), 5-FU (50 µM), AZD1152 (50 nM) or the sequential combination of the drugs. A minimum of four tissue sections from each tumour were split over each treatment group. Following treatment, slices of each group were fixed in 10% neutral buffered formalin and processed as per routine clinical specimens for immunohistochemistry.

### Immunohistochemistry

Immunohistochemistry was performed using both manual (Novolink Polymer Detection Kit (Leica)) and automated (using Ventana Ultra) methods. For manual staining, briefly, slides were deparaffinised, rehydrated, washed, and quenched. Tissue sections underwent antigen retrieval in EDTA buffer (Sigma-Aldrich) at pH 8.5 using a Decloaking Chamber (Biocare Medical). Staining for Ki67 (Cell Signaling #9027) and cleaved caspase 3 (Asp175) (Cell Signaling #9661) was performed by incubating the slides in primary antibody respectively diluted 1:100 in Da Vinci Green (Biocare Medical (MetaGene, Redcliffe, Australia)) with overnight incubation. Antibody-Antigen complexes were detected by 3, 3’-diaminobenzidine (DAB) and counterstained with hematoxylin. Positive controls relevant for the IHC stain of interest were utilized.

### Bioinformatics, statistical analysis and reproducibility

Statistically significant proteins from the comparison of 5-FU treated cells versus untreated cells were subjected to pathway analysis that was performed using the Reactome tool [19], gene ontology (GO) process analysis and the Wikipathways tool. For protein network analysis, all proteins at a q-value threshold ≤ 0.1 were selected for StringDB analysis. Network was generated with default confidence (medium confidence, 0.4) and evidence from active interaction sources included, text mining, experiments, databases, co-expression, neighborhood, gene fusion, and co-occurrence.

Statistical analysis was conducted using GraphPad Prism V9 software. Results are displayed as mean ± SD from at least 3 independent experiments. Distribution was assessed and statistical significance was determined using a Kruskal–Wallis one-way ANOVA test. *P* values below 0.05 were considered significant.

## Results

### Identification of proteins and cellular pathways deregulated by 5-FU in CRC cell lines

**Fig. 1:**
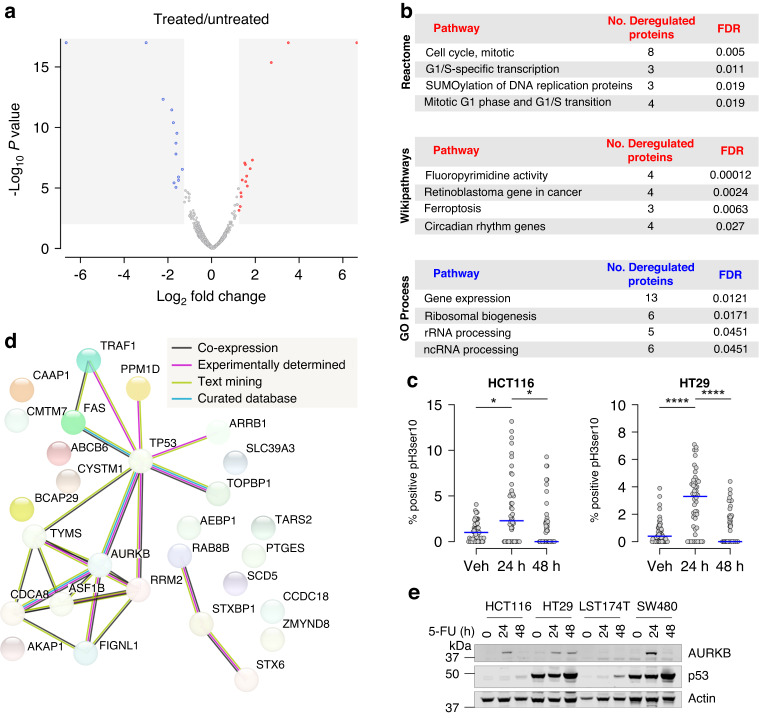
Identification of proteins and pathways that are deregulated by 5-FU. **a** Volcano scatter plot of log2 fold protein changes (5-FU treatment versus untreated) ranked by significance (-log_10_ *P* value) with proteins of *q*-value < 0.1 highlighted in grey sections. **b** List of top four pathways deregulated by 5-FU ranked by FDR. Upregulated pathways identified by (*upper*) Reactome analysis and (*middle*) Wikipathways are indicated in red. Downregulated pathways identified by (*lower*) GO biological process are indicated in blue. **c** Beeswarm plot showing the mitotic index determined by histone pH3Ser10 staining and high throughput immunofluorescence microscopy of (*left*) HCT116 cells and (*right*) HT29 cells treated with 5-FU over 48 h. Data points represent an average percentage of mitotic nuclei per field of view from a minimum of 1100 nuclei (*n* = 23 fields total). Blue lines indicate median values. (ANOVA Kruskal–Wallis multiple comparisons, *p < 0.05, ****p < 0.0001). **d** StringDB network analysis of proteins identified by quantitative proteomics as significantly upregulated following treatment with 5-FU. Lines linking nodes is indicative of known or predicted interactions and associations between identified proteins. **e** Western blot analysis of endogenous AURKB and p53 protein from lysates of CRC cell lines treated in the absence or presence of 5-FU for 24 and 48 h. α-Actin used as loading control.

To identify proteins significantly deregulated by 5-FU in CRC, quantitative proteomics was performed on whole cell lysates of HCT116 cells either untreated or following a 24 h treatment of 5-FU. This cell line was selected as a commonly utilised CRC cell line for in vitro assays. We have previously employed this approach in non-small cell lung cancer (NSCLC) cells to identify proteins deregulated by chemotherapeutic agents that could be exploitable to improve sensitivity to chemotherapy [20]. Quantitative proteomics identified 3590 proteins, whereby 1512 proteins were downregulated by 5-FU treatment and 2078 proteins were upregulated (Supplemental Table 1). Of these proteins, 5-FU significantly induced the downregulation of 30 proteins and upregulation of 29 proteins (Fig. 1a). The top three 5-FU-induced downregulated proteins based upon significance and log2 fold change were queuine tRNA-ribosyltransferase catalytic subunit 1 (QTRT1), NEDD4-like E3 ubiquitin protein ligase (NEDD4L) and epsin 1 (EPN1) (Supplemental Table 1). The top three upregulated proteins were syntaxin 6 (STX6), stearoyl- CoA desaturase 5 (SCD5) and paralog of XRCC5 and XLF (PAXX, formerly C9orf142), each of which have been associated with roles in CRC or other solid malignancies [21–24].

We next sought to determine the molecular pathways in CRC that are impacted by 5-FU by subjecting the identified significantly up- and downregulated proteins (FDR ≤ 0.05) to comparative pathway analysis using the tools Reactome, Wikipathways and GO biological process. As shown in Fig. 1b, Reactome analysis identified pathways for upregulated proteins with roles in regulation of G1, S and mitotic cell cycle progression, cell cycle-specific transcription and control of DNA replication. To complement these analyses, the Wikipathways analysis tool was also investigated which identified roles for these upregulated proteins within the pathways of fluoropyrimidine activity, regulation of G1/S transition (via the retinoblastoma (Rb)) and ferroptosis. While these pathway analysis tools did not identify pathways considered as significant for those proteins downregulated by 5-FU, GO biological process analysis did indicate downregulated pathways impacted by 5-FU included modulation of gene expression, ribosomal biogenesis, and processing of ribosomal and non-coding RNAs. These findings are consistent with the known functions and mechanisms of action for 5-FU, whereby this agent disrupts RNA synthesis and processing, and cell cycle progression, as a result of disrupted DNA replication [25].

Having abundantly detected pathways within those proteins upregulated by 5-FU, and those associated with cell cycle progression, we next sought to validate these findings in two CRC cell lines. As mitotic progression was a prominently identified pathway, the mitotic index of HCT116 and HT29 cells treated with or without 5-FU over 48 h was investigated by performing high content immunofluorescence of pH3Ser10, a marker of mitotic cells. Consistent with the pathway analysis, 5-FU significantly increased the detection of cells accumulating in mitosis at 24 h by ~2-fold in HCT116 cells (*p* = 0.041) and ~8-fold in HT29 cells (*p* < 0.0001; Fig. 1c). Whereas at 48 h, the mitotic index was markedly reduced in both cell lines, pointing to the possibility that cells had exited mitosis. Taken together, our findings that increased mitotic index (detectable pH3ser10) following 24 h exposure to 5-FU are consistent with the quantitative proteomics pathway analysis which identified upregulated mitotic and cell cycle-related pathways.

To further investigate the functional importance of the proteins upregulated by 5-FU, the significantly deregulated proteins were subjected to STRINGdb analysis which complements the previously described pathway tools by evaluating the potential networks of protein-protein interactions between the identified proteins [26]. As shown in Fig. 1d, these analyses identified network clusters between 52% of the proteins significantly upregulated by 5-FU treatment. p53 and AURKB were identified as two core proteins within these networks. AURKB is reported to phosphorylate and modulate levels of p53 [27]. Indeed, these proteins are functionally linked with cell cycle progression and cell survival pathways, especially within G2 and mitosis [28, 29] and, at least for p53, associated with the CRC cell response to 5-FU [30, 31]. To validate the quantitative MS and analysis, western blot analysis to assess p53 and AURKB protein levels was performed on whole cell lysates collected from a small panel of CRC cell lines treated with 5-FU over 48 h. As shown in Fig. 1e, AURKB protein was markedly upregulated following 24 h of treatment with 5-FU in HCT116, HT29 and SW480 cell lines. p53 protein was robustly detected in HT29 and SW480 cells where 5-FU induced upregulation of p53 in all cell lines, predominantly at 48 h following commencement of treatment. These data suggest that 5-FU treatment upregulates both p53 and AURKB proteins in CRC cells.

### Combining the AURKB inhibitor AZD1152 with 5-FU

**Fig. 2:**
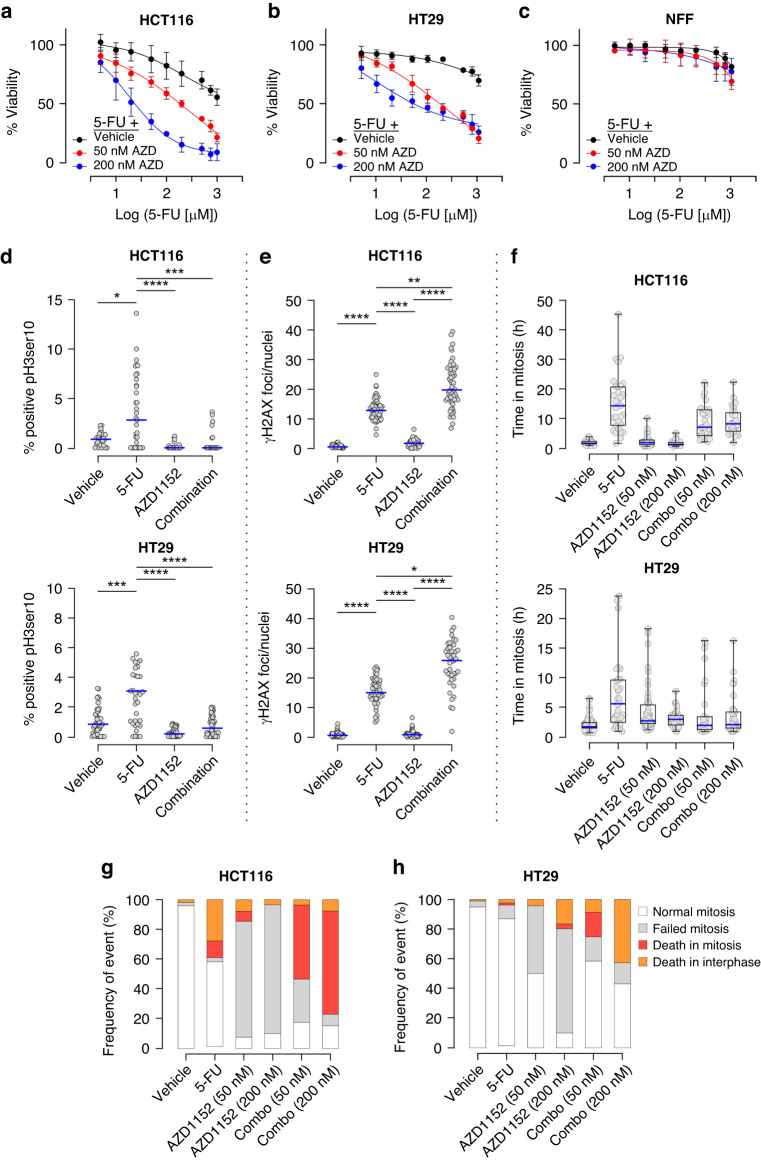
Sequential combination of 5-FU and AZD1152 enhances CRC cell line sensitivity to chemotherapy in 2D. **a**–**c** Dose response curves for escalating concentrations of 5-FU alone (black line) and showing the impact of sequentially combining a low (50 nM; red line) or high (200 nM; blue line) concentration of AZD1152 with 5-FU in (**a**) HCT116 cells, (**b**) HT29 cells and (**c**) neonatal foreskin fibroblast (NFF) cells. *n* = 3. **d** Beeswarm plot showing the mitotic index determined by histone pH3Ser10 staining and high throughput immunofluorescence microscopy of (*upper*) HCT116 cells and (*lower*) HT29 cells treated in the absence or presence of 5-FU, AZD116 or the sequential combination of both drugs. Data points represent an average percentage of mitotic nuclei per field of view from a minimum of 1100 nuclei (*n* = 23 fields total). Blue lines indicate median values. (ANOVA Kruskal–Wallis multiple comparisons, *p < 0.05, ***p < 0.0005, ****p < 0.0001). **e** Beeswarm plots showing the foci count per nucleus of γH2AX immunofluorescence microscopy for (*upper*) HCT116 and (*lower*) HT29 cell lines either untreated, 5-FU or AZD1152 treated for 24 h or treated sequentially with both drugs. Data points represent an average of γH2AX foci/nuclei per field of view from a minimum of 1100 nuclei (n = 23 fields total). Blue lines indicate median values. (ANOVA Kruskal–Wallis multiple comparisons, *p = 0.05, **p = 0.0037, ****p < 0.0001). **f** Box and whisker *p*lots showing the duration of mitosis (between mitotic entry and exit) for individual (*upper*) HCT116 or (*lower*) HT29 cells tracked by live cell imaging. Cells were treated vehicle, 5-FU alone, AZD1152 (50 nM or 200 nM) alone, or sequential combination of 5-FU and low or high concentrations of AZD1152. Blue lines indicate median values. A minimum of 50 cells were tracked. *n* = 3. **g**, **h** The fate of (**g**) HCT116 and (**h**) HT29 cells treated with vehicle, 5-FU alone, AZD1152 (50 nM or 200 nM) alone, or sequential combination of 5-FU and low or high concentrations of AZD1152 were tracked by live cell imaging (minimum of 50 cells) over 72 h and imaged every 15 min.

Having identified key proteins and pathways that are upregulated in response to 5-FU, we postulated that pharmacological targeting of druggable deregulated proteins and those within identified networks would enhance sensitivity to 5-FU. Of these proteins, AURKB is druggable and has been the focus of drug development and clinical trials [32]. To examine this possibility that targeting AURKB might enhance sensitivity to 5-FU, we evaluated the combination of 5-FU with the clinically tested AURKB inhibitor AZD1152. Two concentrations of AZD1152 were tested based upon prior studies [33–35]. Initial combination of either a low (50 nM) or high concentration (200 nM) of AZD1152 with escalating concentrations of 5-FU did not enhance 5-FU potency (Supplemental Fig. 1A, B). Flow cytometry analysis confirmed these individual concentrations of AZD1152 were capable of inducing polyploidy ( > 4 N) in each cell line evaluated (Supplemental Fig. 2A, B). As we observed an upregulation of AURKB following an 24 h exposure to 5-FU, we investigated a sequential combination strategy whereby cells were treated with AZD1152 following a 24 h treatment with 5-FU. As shown in Fig. 2a, b, addition of 50 nM or 200 nM of AZD1152 markedly improved sensitivity to 5-FU, with the higher concentration of AZD1152 improving 5-FU potency ~12-fold and ~82-fold in HCT116 and HT29 cells respectively. The sequential combination strategy did not impact 5-FU sensitivity in non-malignant neonatal foreskin fibroblast (NFF) cells (Fig. 2C).

We next sought to examine the impact of this strategy on cell cycle and induction of DNA damage. Compared to vehicle treated HCT116 and HT-29 cells, 24 h 5-FU treatment increased the proportion of cells in G2/M phase for all cell lines except SW480 cells (Supplemental Fig. 2A, B), and the mitotic index of HCT116 and HT29 cells ~3 fold (Fig. 2d). A 24 h AZD1152 treatment abrogated detection of pH3Ser10 positive cells. Sequential combination of 5-FU and AZD1152 blocked the 5-FU-induced increase in mitotic index for both HCT116 (*p* = 0.002) and HT29 cells (*p* = <0.0001). As 5-FU induces genotoxic stress by depleting nucleotide pools and impacting DNA synthesis, which can induce replication stress [25], we examined the DNA damage response by performing high content immunofluorescence and quantifying foci formed by the DNA damage marker γH2AX following treatment. In both HCT116 and HT29 cells, 5-FU induced a ~ 15-fold increase in detectable γH2AX foci (Fig. 2e). While AZD1152 alone did not impact the number of γH2AX foci per nuclei, the sequential strategy further increased the detection of DNA damage compared to 5-FU treatment alone in both cell lines (HCT116 *p* = 0.0037; HT29 *p* = 0.05).

We next employed live cell imaging analysis to track mitoses and stage of cell death on treatment. Using this approach indicated that 5-FU markedly increased the time HCT116 cells spent in mitosis (Fig. 2f). AZD1152 treatment alone did not impact mitosis duration. Combining 5-FU with either a 50 nM or 200 nM concentrations of AZD1152 reduced the time cells spent in mitosis by~2-fold compared with 5-FU alone. Consistently in HT29 cells, 5-FU or AZD1152 treatment alone increased the mitotic time, whereas the sequential strategy reduced the time spent in mitosis by ~2.5-fold for both AZD1152 concentrations, relative to 5-FU treatment alone.

Having observed that the sequential strategy improved 5-FU potency and increased detectable DNA damage, we next used the live cell analysis to investigate cell fate. In HCT116 cells, 24 h 5-FU treatment induced death in interphase while AZD1152 treatment alone, consistent with earlier reports [36], resulted predominantly in failed mitoses (Fig. 2g). The sequential strategy in this cell line resulted in cells predominantly undergoing cell death in mitosis. On the other hand, in HT29 cells, the extent of cell death in interphase and failed mitosis upon 24 h 5-FU treatment and AZD1152 treatments respectively, was less in comparison to that observed in HCT116 cells. Moreover, the sequential strategy in HT29 cells promoted cell death predominantly in interphase, at least with the higher concentration of AZD1152 (Fig. 2h). Accordingly, it is possible that this strategy might sensitise cells to 5-FU via different mechanisms, perhaps in part due to mitotic control, which is in line with the differences seen between cell lines in the time spent in mitosis (Fig. 2f). Nevertheless, these data suggest that the sequential strategy improves 5-FU sensitivity.

### Evaluating sequential drugging of 5-FU and AZD1152 in 3D in vitro models

**Fig. 3:**
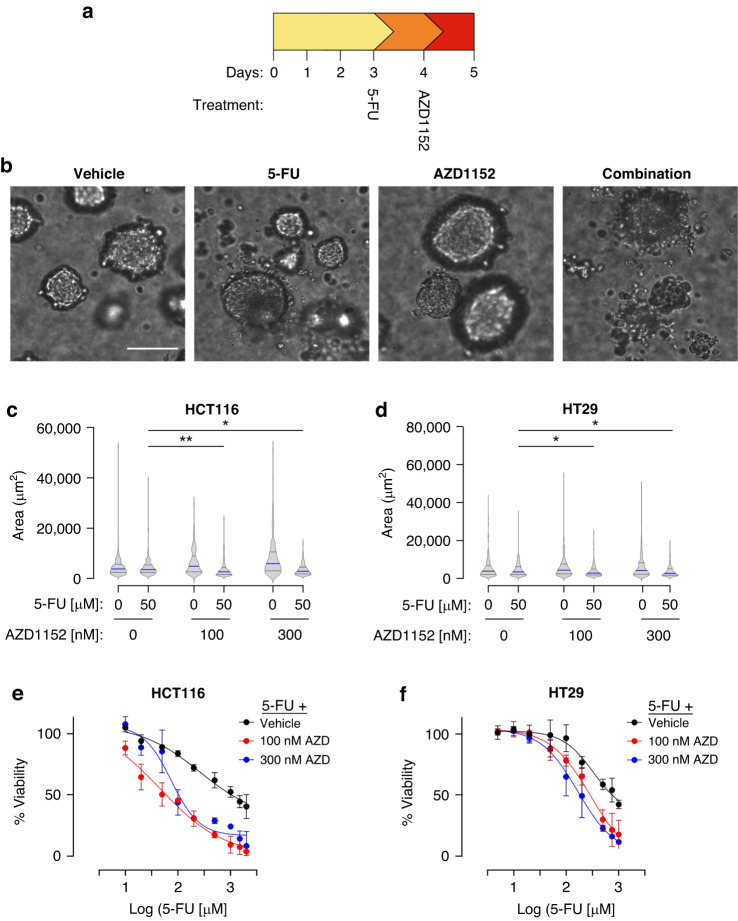
The sequential combination of 5-FU and AZD1152 is effective in CRC cell line 3D models. **a** Sequential treatment schedule of 5-FU and AZD1152 for spheroids of CRC cell lines grown in 3D over 72 h. **b** Representative images of HCT116 spheroids treated with vehicle, 5-FU alone, AZD1152 alone, or sequential combination of 5-FU and AZD1152. Scale bar = 100 µm. **c**, **d** Quantification of spheroid area, calculated using Image J software, from images of (**c**) HCT116 and (**d**) HT29 derived spheroids treated with vehicle (0), 5-FU alone, a low (100 nM) or high (300 nM) concentration of AZD1152 alone, or sequential combination of 5-FU and AZD1152. (ANOVA Kruskal–Wallis multiple comparisons, *p < 0.05, **p < 0.008). **e**, **f** Dose response curves for escalating concentrations of 5-FU alone (black line) and showing the impact of sequentially combining a low (100 nM; red line) or high (300 nM; blue line) concentration of AZD1152 with 5-FU in (**e**) HCT116 cells and (**f**) HT29 cells. *n* = 3.

Following the observation that the sequential strategy enhanced 5-FU sensitivity in 2D cell monolayers, we next sought to evaluate the effectiveness of this approach on 3D spheroid structures. Cell line-based 3D models were generated using Happy Cell ASM 3D medium enabling spheroid growth in suspension. 3D spheroids grown for three days were subjected to either an individual 24 h 5-FU concentration, low or high AZD1152 concentrations, or sequential exposure to both drugs, in line with the treatment strategy used in the 2D models (Fig. 3a). Of these treatments, microscopy imaging revealed that the sequential strategy resulted in spheroid Higher concentrations of drug were selected to account for drug diffusivity [37]. Morphologically, individual treatment of HT29-based structures with 5-FU or AZD1152 did not impact spheroid integrity, while AZD1152 treatment yielded larger spheroids compared with vehicle treated structures (Fig. 3b). Quantification of spheroid area for both HCT116 (Fig. 3c) and HT29 structures (Fig. 3d) confirmed that AZD1152 treatment, especially for the higher concentration, yielded ~1.3-fold and ~1.6-fold increases in spheroid area, for HCT116 and HT29 cells respectively. Unlike individual 5-FU or AZD1152 concentrations, the sequential combination strategy resulted in gross spheroid disintegration while individual 5-FU or AZD1152 did not markedly impact spheroid morphology, compared with vehicle (Fig. 3b). As a result, a significant decrease was observed in spheroid area upon application of the sequential strategy in both HCT116 (Fig. 3c) and HT29 spheroids (Fig. 3d). Evaluation of the sequential strategy on spheroid viability demonstrated that, consistent with 2D models, AZD1152 enhanced 5-FU potency in HCT116 spheroids (Fig. 3e), with the increased potency being more prominent for the higher AZD1152 concentration in HT29 spheroids (~2-fold; Fig. 3f). Taken together, these data suggest that AZD1152 enhances 5-FU sensitivity in 2D and 3D in vitro models of CRC.

### AZD1152 enhances the ex vivo response of patient-derived CRC tumours to 5-FU

**Fig. 4:**
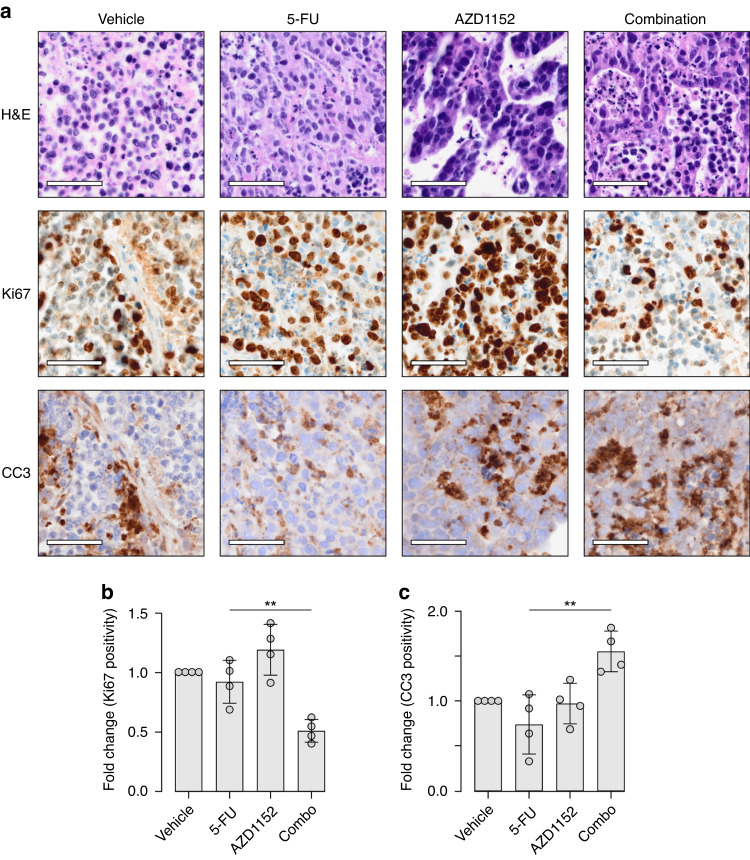
The sequential combination of 5-FU and AZD1152 is effective in ex vivo cultures of patient-derived xenografts of CRC. **a** Representative images of H&E staining and Ki67 and cleaved caspase 3 (CC3) immunohistochemistry staining of tissue slices from a patient-derived CRC adenocarcinoma xenograft grown in vivo, and cultured ex vivo after treatment with vehicle, 5-FU alone, AZD1152 alone or sequential combination of 5-FU and AZD1152. Scale bar = 50 µm. **b**, **c** (**b**) Ki67 and (**c**) CC3 staining quantification of (**a**), calculated using QuPath software, with fold change in cells positive for staining relative to vehicle treated tissue slices. Average of four tissue slices from 3 tumours. (ANOVA Kruskal–Wallis multiple comparisons, **p < 0.004).

Given that AZD1152 improves the sensitivity of 5-FU in 2D and 3D in vitro models of CRC, we next examined the sequential strategy on tissue sections cut from freshly isolated patient-derived xenografts (PDXs) of a CRC. A previously established PDX of a poorly differentiated primary colonic adenocarcinoma [17] was resected and cultured ex vivo. Tissue slices were either subjected to individual concentrations of 5-FU or AZD1152 or the sequential combination of the drugs. Therapy response was assessed by quantification of Ki67, to measure active proliferation, or cleaved caspase 3 (CC3), to measure apoptosis, via immunohistochemistry staining. As shown in Fig. 4a–c, 5-FU treatment alone, relative to vehicle treatment, did not impact Ki67 or CC3 staining. While AZD1152 treatment alone did not induce an increase in CC3, elevated Ki67 staining was detected. While classically considered a cell cycle inhibitor, Phase I trialling of AZD1152 is demonstrated to not reduce levels of the proliferation marker Ki67, likely due to the induction of endoreplication following AURKB inhibition [38]. Unlike the individual treatments, the sequential combination strategy induced a significant reduction in Ki67 staining (*p* = 0.001) and increase in CC3 staining (*p* = 0.0052). These results indicate that the sequential strategy can reduce tumour proliferation and induce tumour cell death. Collectively, these data suggest that AURKB blockade following therapy with 5-FU is a unique strategy to enhance the effectiveness of chemotherapy in CRC.

## Discussion

Poor response to 5-FU-based chemotherapy remains a major issue for treatment failure and a common challenge to improving health outcomes for people living with CRC [39]. To address this global challenge, novel biomarkers and therapeutic strategies are needed to transform the management of CRC. The aim of this study was to employ quantitative proteomics to better understand the response of CRC cells to 5-FU, to identify proteins that are [1] deregulated following a 24 h exposure to 5-FU, and [2] whose function might be exploited to sensitise CRC cells to chemotherapy. We have hypothesised that molecularly profiling the response to 5-FU and drugging novel deregulated targets, will improve the sensitivity of CRC cells to 5-FU. Here, we identified key upregulation of proteins involved in pathways associated with DNA replication, transcription and mitotic progression, largely in keeping with known modes of action for 5-FU [25]. Of these proteins, we identified that 5-FU upregulated AURKB as a central protein within a wider network of upregulated proteins. AURKB is an attractive therapeutic target yielding small molecules in clinical trials (reviewed in [40]). Our findings demonstrate that inhibition of AURKB with AZD1152, in a sequential strategy following 5-FU exposure, improves the sensitivity of CRC cells to 5-FU, and not non-malignant fibroblasts. Whether this approach to enhance 5-FU sensitivity contributes to preventing therapy resistance is a matter for further research.

AURKB is a member of the aurora serine/threonine kinase family and functions as an essential regulator of mitotic progression and chromosomal separation as the catalytic subunit of the chromosomal passenger complex [41–43]. Upregulation or overexpression of AURKB has been established across a variety of human tumours as a contributor to tumorigenesis [44–46]. In CRC, elevated expression of AURKB is significantly associated with a poorer overall survival, providing further merit for inhibition of AURKB as an approach to target CRC. Using in vitro approaches, we identify that 5-FU upregulates the AURKB protein and kinase activity, as marked by phosphorylation of pH3Ser10 (Fig. 1). Increased AURKB activity was noted at 24 h following 5-FU treatment but not at 48 h. While the observed increases in the proportion of G2/M cells is consistent with prior studies [30, 47], the eventual drop in mitotic index at 48 h is likely due to a G1-S phase arrest where 5-FU impacts DNA and RNA synthesis [48]. Furthermore, at 24 h, we demonstrate that 5-FU markedly increases time spent in mitosis (Fig. 2). As such, these findings provide support for the sequential approach of 5-FU treatment followed by AURKB inhibition, where this kinase is temporally active, versus an initial combination strategy.

As an AURKB inhibitor, the small molecule inhibitor AZD1152 (commercial name barasertib) has demonstrated dose-dependent tumour-suppressive and apoptotic activity in multiple myeloma, lung, breast, colorectal and pancreatic cancer cell lines [49–51]. Monotherapeutic clinical testing of AZD1152 (and hydroxyquinazoline pyrazole anilde-AZD1152, designated as AZD2811) has yielded stable disease for a subset of patients with solid malignancies [38]. However, clinical development of AstraZeneca’s AURKB targeting program (AZD1152 and AZD2811) has stalled with the termination of a recent Phase I/II trial (NCT03217838). Although AZD1152 has been demonstrated as tolerable with manageable toxicity profiles in early phase trial, suitability as a monotherapy outside of preclinical models has not been established. While predictive markers for patient selection might help guide continued clinical development [52], we propose that identifying improved combinations and treatment strategies would reinvigorate clinical development and, with further preclinical testing, enable optimal design of further trials to maximise the clinical efforts.

To improve therapeutic outcomes, several combination strategies have been investigated with barasertib (AZD1152 and AZD2811). For example, combining barasertib with selumetinib, a MEK1/2 inhibitor, demonstrated enhanced efficacy in human xenograft models [53]. For chemotherapeutic agents, combining barasertib with cytarabine increased cytotoxicity in acute myeloid leukaemia cells [54]. Similarly, in solid malignancies, barasertib has enhanced efficacy of paclitaxel, CPT-11, irinotecan, gemcitabine and oxaliplatin [33, 55, 56]. In these studies, AZD1152 was optimally employed as a pre-treatment to gemcitabine or oxaliplatin, including in a CRC setting, to sensitise to chemotherapy [33]. With polyploidy induced by AURKB inhibition, it is possible that the potency of these chemotherapeutics was greater in cells undergoing endoreplication. Indeed, in our hands AZD1152 alone resulted in failed mitoses and prominent multinucleation (Fig. 2g, h). However, the order of sequential combination differs to our study, where we rationally employed AZD1152 following upregulation of AURKB activity, where this inhibitor markedly sensitised cells with prior 5-FU treatment in in vitro and ex vivo models of CRC. Notwithstanding, a possible explanation for this difference may lie in varying mechanisms of action for the individual chemotherapeutic agents, that is to compare platinum-based with fluoropyrimidine agents. However, to our knowledge, we are the first to investigate a combination between AZD1152 and 5-FU.

In our study, the sequential combination strategy was effective at improving 5-FU sensitivity in two CRC cell lines HT29 and HCT116, while not in non-malignant fibroblast cells (Fig. 2). Mechanistically, this strategy markedly reduced mitotic index and time in mitosis, relative to 5-FU alone, while levels of the DNA damage marker γH2AX was significantly elevated. However, the stage of cell death differed between the two cell lines. HCT116 cells underwent mitotic cell death whereas HT29 cells, while partially capable of completing mitosis, underwent death in interphase, predominantly in those cells treated with a combination including higher AZD1152 concentrations (Fig. 2g, h). A key point of difference between these lines is wildtype (HCT116) versus mutant (HT29) p53. AURKB inhibition in p53 wildtype cells is reported to trigger mitotic slippage whereas the eventual cellular outcome for p53 deficient cells is cell death via chromosomal instability resulting from multipolar mitosis failure and genome endoreplication [57]. Like AZD1152, 5-FU was also recently reported to be more effective in p53 deficient patient derived organoid models of CRC [58]. This is suggested to be due to a failure of p53-deficient cells to halt proliferation following 5-FU-induced DNA damage. Consistent with recent reports [58], our proteomics of p53 wildtype HCT116 cells noted the 5-FU-induced upregulation of p53 protein (Fig. 1). In keeping with this observation and earlier reports [58], we note that 5-FU alone predominantly induced cell death during interphase (Fig. 2g), pointing to a functional G1 checkpoint within these cells. Nonetheless, irrespective of p53 status, the sequential combination of 5-FU and AZD1152 was effective at enhancing the sensitivity of cells to 5-FU. Indeed, this enhanced response was further observed ex vivo in a patient derived CRC (Fig. 4). Nevertheless, further studies are warranted to examine the complex interplay between the tumoral genetic landscape and mechanistic induction of cell death.

Our exploratory study has identified a possible novel sequential combination strategy to improve sensitivity of CRC to chemotherapy. Further studies are needed to tease out the underlying mechanisms supporting how this combination strategy functions. We speculate that the temporal juncture between 5-FU induced replication stress and AURKB inhibition, which induces endoreplication [59], promotes cancer cell death. However, while novel, there are several limitations with our study. It is worth noting that our proteomics approach only employed a single cell line which is not representative of all CRC tumours. Further proteomics analysis of the 5-FU response in in vitro or patient derived CRC models would strengthen our findings, especially to examine chemotherapy response in tumours with common CRC mutations such as mutant p53 and KRAS. Indeed, additional testing of our sequential combination in a wider panel of CRC models, including in vivo, is needed to strengthen our findings herein. Nonetheless, our study employing 2D and 3D in vitro models and ex vivo testing point to the utility of sequentially combining 5-FU and AURKB inhibitors in CRC. Moreover, the drug concentrations used within out study are also within the known maximum tolerated doses of 5-FU [60] and for AZD1152, namely the lower concentration, which was examined in Phase I trial [50]. Further research is required to determine whether this strategy to enhance sensitivity to 5-FU ultimately prevents resistance to 5-FU in colorectal tumours. Although our findings will not immediately influence clinical decision making or therapy development, our work warrants further investigation as a strategy to improve the efficacy of 5-FU for CRC.

### Supplementary information

Supplemental material figure legends

Supplemental Figure 1

Supplemental Figure 2

Supplemental uncropped western blots

Supplemental Table 1

## Data Availability

All data that supports the findings of this study are included in this published article and available in the Supporting Information Material of this article.
