# Supplementary material for: Inhibition of Aurora B kinase (AURKB) enhances the effectiveness of 5-fluorouracil chemotherapy against colorectal cancer cells
Source: Br J Cancer. 2024 Jan 29;130(7):1196–205. doi: 10.1038/s41416-024-02584-z (PMC10991355; doi:10.1038/s41416-024-02584-z)
Supplement: Supplementary file 1 — Supplemental material figure legends [file 41416_2024_2584_MOESM1_ESM.docx]

**Supplemental Fig. 1** Dose response curves for escalating concentrations of 5-FU alone (black line) and showing the impact of an initial combination of low (50 nM; red line) or high (200 nM; blue line) doses of AZD1152 with 5-FU in (A) HCT116 cells, (B) HT29 cells. n = 3.

**Supplemental Fig. 2** (A) Representative DNA content cell cycle histogram for vehicle, 5-FU and AZD1152 (50 nM and 200 nM) treated CRC cells (HCT116 cells). (B) Stacked column plot showing the proportion of cell cycle stage quantified from the DNA content flow cytometry of four CRC cell lines treated with vehicle, 5-FU and 50 nM or 200 nM of AZD1152 for 24 h.
