# Supplementary figures and images for: Inhibition of Aurora B kinase (AURKB) enhances the effectiveness of 5-fluorouracil chemotherapy against colorectal cancer cells
Source: Br J Cancer. 2024 Jan 29;130(7):1196–205. doi: 10.1038/s41416-024-02584-z (PMC10991355; doi:10.1038/s41416-024-02584-z)

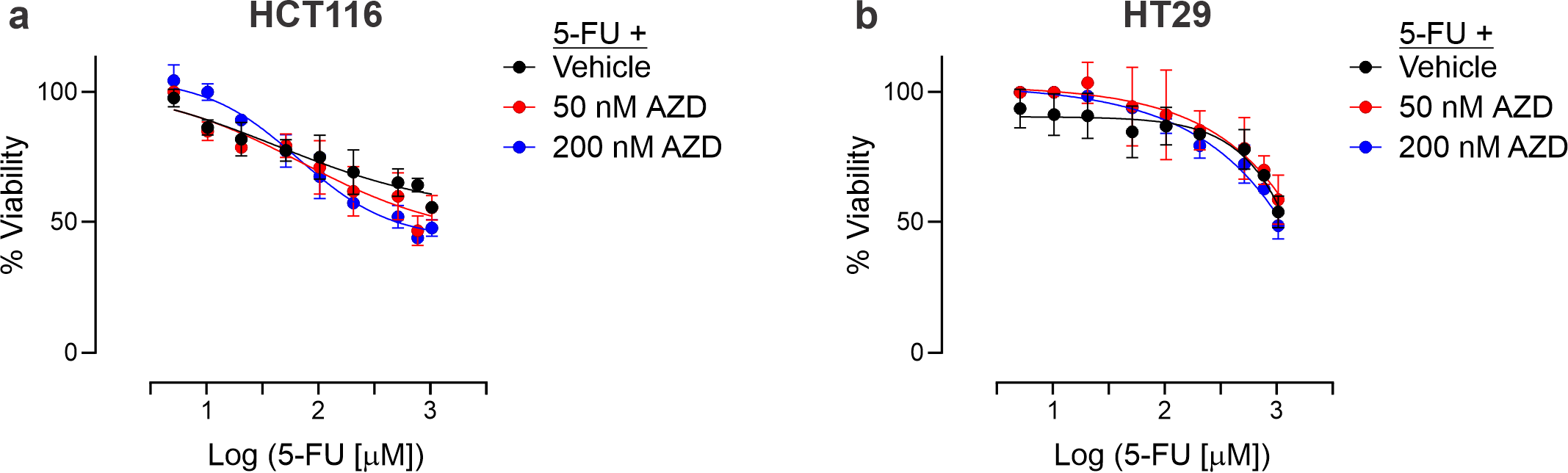

Supplement: Supplementary file 2 — Supplemental Figure 1 [file 41416_2024_2584_MOESM2_ESM.tif]

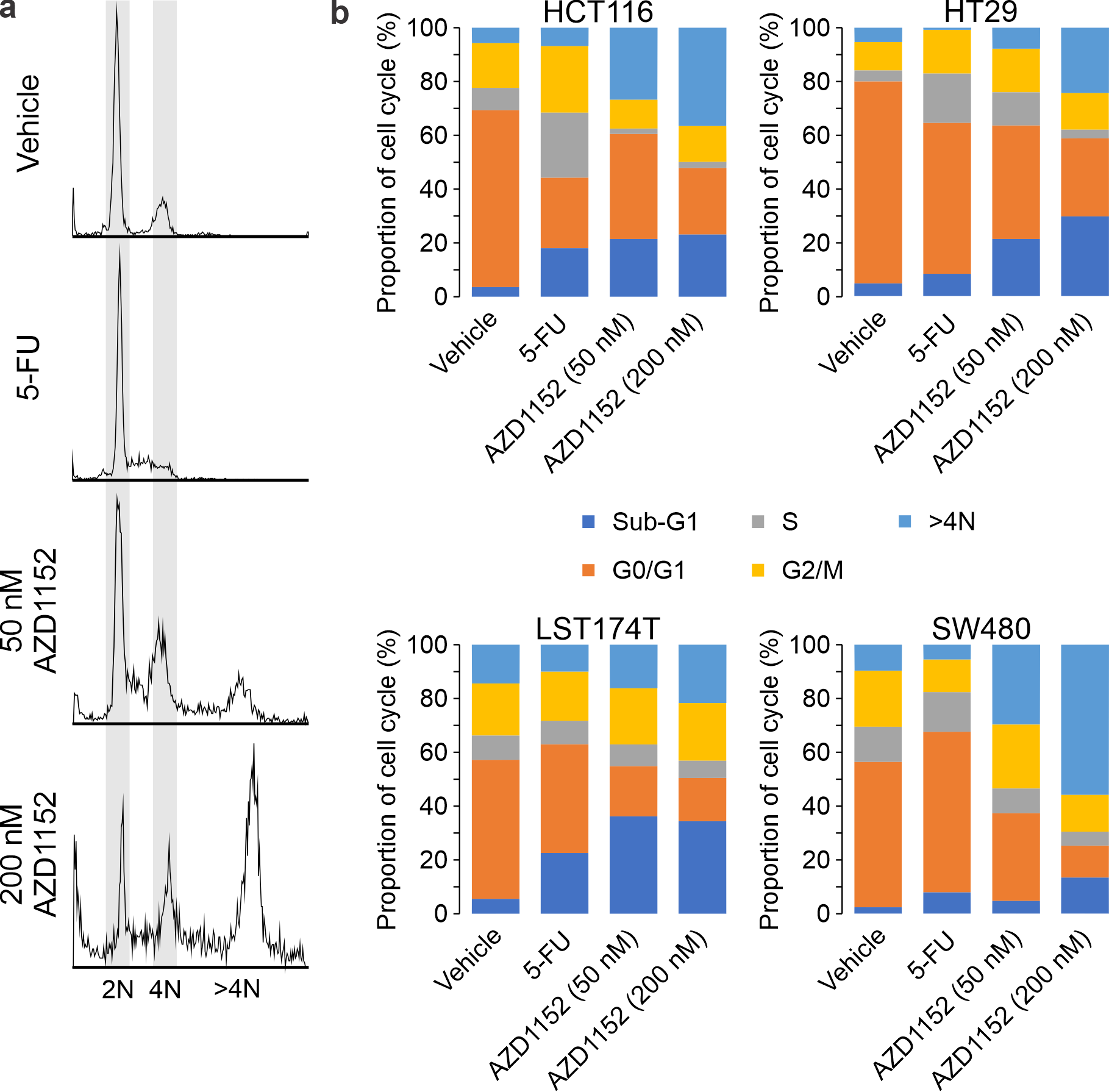

Supplement: Supplementary file 3 — Supplemental Figure 2 [file 41416_2024_2584_MOESM3_ESM.tif]

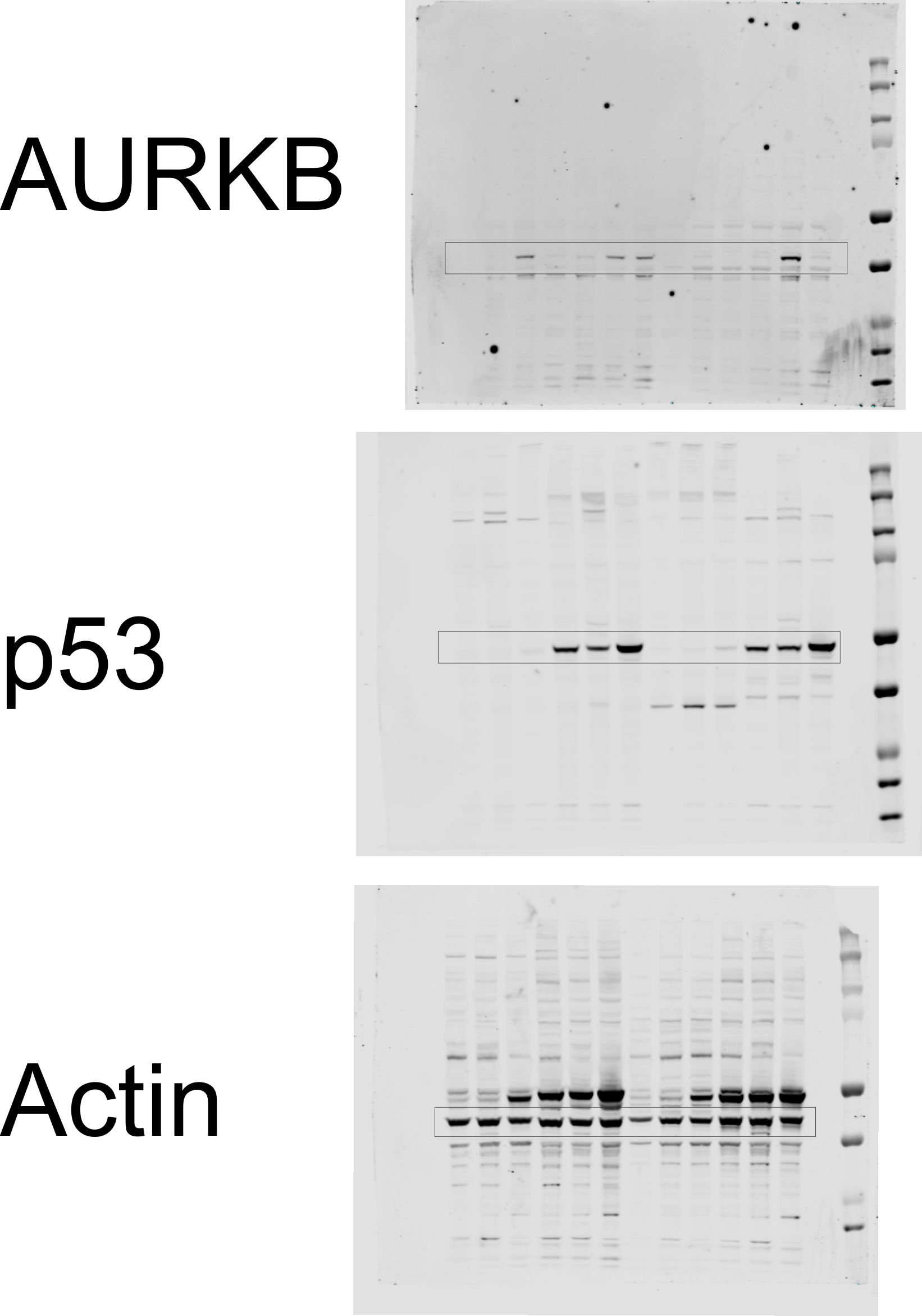

Supplement: Supplementary file 4 — Supplemental uncropped western blots [file 41416_2024_2584_MOESM4_ESM.jpg]
